# Durable remission of primary testicular diffuse large B-cell lymphoma with secondary cutaneous involvement with chimeric antigen receptor T-cell therapy

**DOI:** 10.1016/j.jdcr.2025.12.046

**Published:** 2026-01-10

**Authors:** Nicole Trepanowski, Robert E. LeBlanc, Caron A. Jacobson, Joi B. Carter, Frederick Lansigan

**Affiliations:** a Department of Dermatology, Dartmouth-Hitchcock Medical Center, Lebanon, New Hampshire; b Geisel School of Medicine at Dartmouth, Hanover, New Hampshire; c Department of Pathology and Laboratory Medicine, Dartmouth-Hitchcock Medical Center, Lebanon, New Hampshire; d Department of Medical Oncology, Dana-Farber Cancer Institute, Boston, Massachusetts; e Dartmouth Cancer Center, Dartmouth-Hitchcock Medical Center, Lebanon, New Hampshire

**Keywords:** CAR T-cell, chimeric antigen receptor, cutaneous, diffuse large B-cell lymphoma, DLBCL, primary, refractory, relapsed, remission, secondary, testicular

## Introduction

Diffuse large B-cell lymphoma (DLBCL) can arise in the skin (primary cutaneous) or be systemic with skin involvement (secondary cutaneous). Both primary and secondary cutaneous DLBCL have a predilection for the lower limbs; however, secondary cutaneous DLBCL is more commonly associated with advanced stage and worse prognosis than primary cutaneous DLBCL, with 5-year overall survival of 31% compared to 65% for primary cutaneous DLBCL.^1^^,^^2^ Ultimately, new treatments are needed to improve the morbidity and mortality associated with secondary cutaneous DLBCL.

Herein, we describe the case of a patient with chemorefractory primary testicular DLBCL with secondary cutaneous involvement who achieved durable remission with chimeric antigen receptor (CAR) T-cell therapy, highlighting the potential of CAR T-cell therapy for this rare condition.

## Case report

A 53-year-old male presented with 3 months of left leg skin changes. He noted progressive hardening, “lumpy” appearance, and discoloration of the left lower leg with associated pain. Additionally, within the last month, he had developed an open wound on the left medial ankle, which was debrided by vascular surgery. Concurrently, he was experiencing left testicular swelling for which he underwent left orchiectomy the week prior. He denied fevers, night sweats, or unintentional weight loss. Testicular pathology was pending at presentation to dermatology.

**Fig 1:**
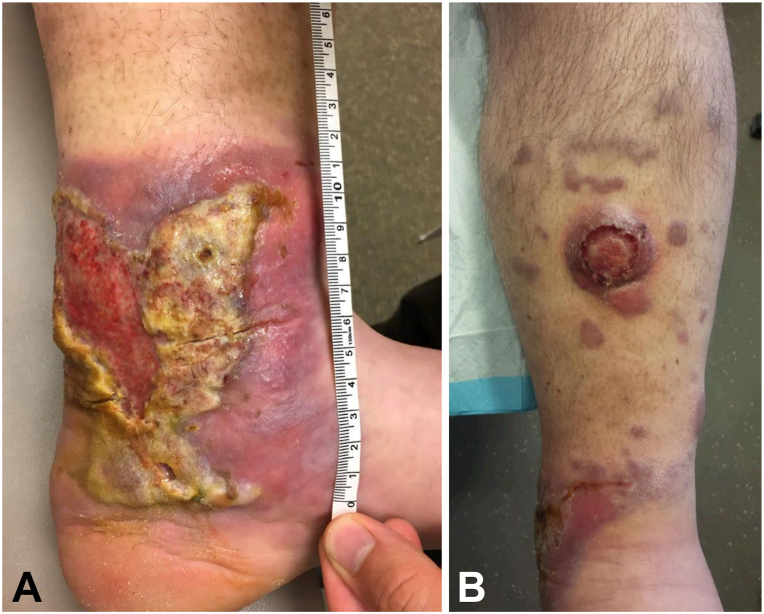
A 12 × 18 cm indurated, ulcerated, tender violaceous plaque on the left medial ankle with fibrinous exudate **(A)** and a 5 cm indurated, ulcerated, *round*, violaceous tumor on the left anterior shin with multiple indurated 1-2 cm well-demarcated, violaceous nodules on the left lower leg **(B)**.

**Fig 2:**
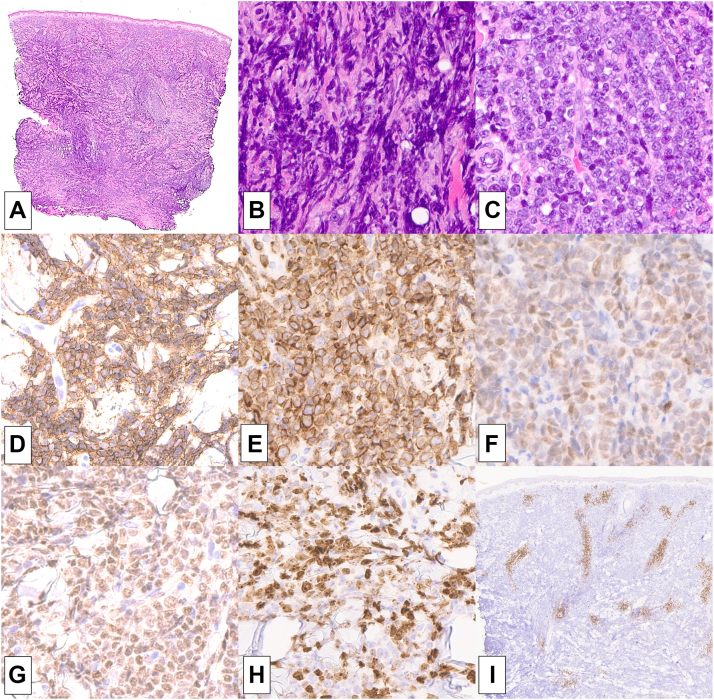
Punch biopsy of diffuse large B-cell lymphoma: The dermis and subcutis are replaced by a dense interstitial infiltrate best appreciated on low power (**A,** H&E 10×). While many of the fine cytomorphologic details are heavily obscured by crush artifact resulting in blurring of cellular boundaries and nuclear details (**B,** H&E 400×), the better preserved areas show monotonous immunoblasts (**C,** H&E 400×). A vast majority of lesional cells are CD20 positive (**D,** 400×), BCL2 positive (**E,** 400×), MUM1 positive (**F,** 400x), MYC positive (**G,** 400×), and Ki-67 positive (**H,** 400×). CD3 highlights relatively sparse, *small T-cells* adjacent to blood vessels and not within the dense B-cell areas (**I,** 20×).

Examination was notable for a 12 × 18 cm indurated, ulcerated, tender violaceous plaque on the left medial ankle with fibrinous exudate, a 5 cm indurated, ulcerated, round, violaceous tumor on the left anterior shin, and multiple indurated 1-2 cm violaceous nodules on the left lower leg (Fig 1). He had no cervical, axillary, or inguinal lymphadenopathy. A 6 mm punch biopsy of the left anterior shin revealed a diffuse dermal and subcutaneous infiltrate of monomorphous immunoblasts. Immunohistochemistry revealed B-cells with an activated cell phenotype. They expressed CD20, MUM1, and BLC2, and were negative for CD10, BCL6, EBER, and Cyclin D1 (Fig 2). Ki-67 highlighted a vast preponderance of the cells and MYC showed moderate intensity expression in approximately 80% of the infiltrate. Pathology slides from the orchiectomy specimen revealed an essentially identical infiltrate. Fluorescence in situ hybridization testing of the skin and testicular samples revealed MYC rearrangement with no BCL2 or BCL6 rearrangement; however, extra copies of BCL2 and BCL6 were identified. Peripheral blood flow cytometry and bone marrow biopsy were negative for leukemic involvement. 18F-fluorodeoxyglucose positron emission tomography combined with computed tomography demonstrated hypermetabolic lesions on the left calf, ankle, and foot. Ultimately, the patient was diagnosed with primary testicular DLBCL of nongerminal center origin with secondary cutaneous involvement.

**Fig 3:**
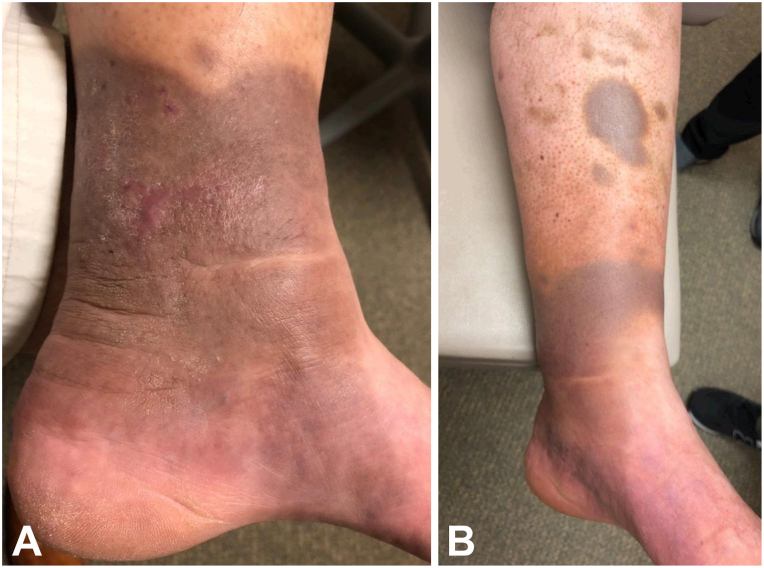
Two months **(A)** and 3 years **(B)** post-treatment with chimeric antigen receptor (CAR) T-cell therapy revealing resolution of lesions on the left lower leg with post-inflammatory hyperpigmentation.

Treatment consisted of 1 cycle of chemotherapy with rituximab, cyclophosphamide, doxorubicin, vincristine, and prednisone followed by 2 cycles of rituximab, cyclophosphamide, vincristine, doxorubicin, and methotrexate and rituximab, ifosfamide, etoposide, and cytarabines. He subsequently received radiation to the right testis and scrotum. Three months after his last chemotherapy treatment, his lymphoma relapsed in the skin of his left lower leg, and he underwent 4 cycles of chemotherapy with an experimental regimen of rituximab, ifosfamide, carboplatin, etoposide, and lenalidomide, with the last cycle without lenalidomide. Due to persistent refractory secondary cutaneous disease, a left lower extremity amputation was considered as a bridge to allogeneic stem cell transplantation; however, the patient ultimately pursued CD19-directed CAR T-cell therapy with axicabtagene ciloleucel. Fludarabine and cyclophosphamide were administered for lymphodepletion. His course was complicated by grade 1 cytokine release syndrome and grade 2 immune-effector cell-associated neurotoxicity syndrome, treated with levetiracetam and systemic steroids. His refractory secondary cutaneous lymphoma rapidly cleared within 1 month of receiving CAR T-cell therapy. He has had no cancer recurrence 7 years post-treatment (Fig 3), though his course has been complicated by prolonged B-cell aplasia, hypogammaglobulinemia, and recurrent sinus infections.

## Discussion

Our case has several unique features. Multiple cutaneous lesions and early cutaneous dissemination within 6 months of diagnosis are associated with worse overall survival in patients with secondary cutaneous DLBCL.^2^ Both the testis and the skin are uncommon sites of extranodal involvement in DLBCL, occurring in 1% or fewer of DLBCL cases at diagnosis, respectively.^3^^,^^4^ Concurrent primary testicular DLBCL with skin and soft tissue involvement at time of diagnosis is even rarer, impacting only 4% of primary testicular DLBCL cases.^4^

Differentiating between primary testicular DLBCL with secondary cutaneous involvement and primary cutaneous DLBCL with secondary testicular involvement can be a diagnostic challenge given these conditions have overlapping clinical, histopathologic, immunophenotypic, and molecular features. Customarily, the distinction is based on the presence of extracutaneous involvement at the time of diagnosis.^5^ Men with primary cutaneous DLBCL should therefore be screened for testicular involvement at diagnosis.^5^

Only 1 other report describes outcomes of primary testicular DLBCL with secondary cutaneous involvement treated with CAR T-cell therapy.^6^ While Adeuyan et al’s case also resulted in flattening of cutaneous lymphoma, follow-up duration and type of CAR T-cell therapy used were not reported.^6^ George et al reported a case of a patient with primary central nervous system (CNS) DLBCL who developed clonally unrelated primary testicular DLBCL.^7^ The patient subsequently developed cutaneous involvement, although clonality testing to determine CNS or testicular origin was not performed.^7^ In that case, the patient ultimately developed progression of disease after treatment with CAR T-cell therapy.^7^ Other cases of primary testicular DLBCL treated with CAR T-cell therapy had mixed results, although those cases were complicated by CNS involvement.^8^^,^^9^ Post-approval, DLBCL patients treated with axicabtagene ciloleucel had an overall response rate of 70% with a complete response of 52% and partial response of 18%.^10^ Our case highlights the potential for durable remission with CAR T-cell therapy for relapsed/refractory DLBCL involving extranodal sites such as skin. Clinicians should be aware of B-cell aplasia as a transient, and in rare instances, a long-term complication of CAR T-cell therapy, indicating persistent CAR T-cell activity.

## Conflicts of interest

None disclosed.
